# Predicting Evaluations of Essay by Computational Graph-Based Features

**DOI:** 10.3389/fpsyg.2020.531262

**Published:** 2020-11-12

**Authors:** Liping Yang, Tao Xin, Canxi Cao

**Affiliations:** Collaborative Innovation Center of Assessment Toward Basic Education Quality, Beijing Normal University, Beijing, China

**Keywords:** automatic essay scoring, writing ability, graph-based features, scoring rubric, reliability

## Abstract

How to effectively evaluate students’ essays based on a series of relatively objective writing criteria has always been a topic of discussion. With the development of automatic essay scoring, a key question is whether the writing quality can be evaluated systematically based on the scoring rubric. To address this issue, we used an innovative set of graph-based features to predict the quality of Chinese middle school students’ essays. These features are divided into four sub-dimensions: basic characteristics, main idea, essay content, and essay development. The results show that graph-based features were significantly better at predicting human essay scores than the baseline features. This indicates that graph-based features can be used to reliably and systematically evaluate the quality of an essay based on the scoring rubric, and it can be used as an alternative tool to replace or supplement human evaluation.

## Introduction

Writing is a highly comprehensive and creative language ability that can express an author’s thoughts and is scored by standardized assessment. For this assessment, an author utilizes their language knowledge and writing strategies to complete a writing task. A high-quality essay has a clear and focused position, effective generation, and organization of ideas, appropriate usage of explications to support the main idea, and well-developed topics.

Automated essay scoring (AES) is the process of scoring and evaluating essays using computer programs (Shermis and Burstein, 2003). Scoring based on computational methods does not depend on a rater’s subjective experience and can simultaneously evaluate and score multiple essay traits and generate a holistic score (Attali and Powers, 2009; Burstein et al., 2013; Ramineni and Williamson, 2013), which improves the objectivity of essay scoring.

The field of AES began with PEG (Project essay grader, Page, 1966), which was developed by Ellis Batten Page and his colleagues in the 1960s. AES systems have integrated insights from multiple disciplines, including psychometrics, computational linguistics, and writing research to score large-scale examinations, such as the e-rater (Burstein et al., 2013) used for the American Graduate Entrance Examination (GRE), the Test of English as a Foreign Language (TOEFL), and the Graduate Management Admissions Test (GMAT). Because the development of AES is always based on the evaluation criteria or scoring rubric of a certain assessment project, the automatic evaluation system is analyzable and stable compared with human scoring. Thus, it has shown great potential to indicate the direction for improvement of students’ writing ability. However, most AES systems are not sufficient in scoring essay ideas and content, which are important evaluation dimensions in writing criteria or scoring rubric of essays.

In this study, we propose a graph-based^1^ text analysis technique to enhance existing state-of-the-art AES systems by incorporating novel features for measuring an essay’s main idea, content, and development. Graph-based text analysis offers further possibilities for AES because of previously demonstrated close relation with text quality (Ke et al., 2016; Somasundaran et al., 2016; Zupanc and Bosnic, 2017). However, the features used in these studies are relatively simple and fragmented, and few studies, especially pertaining to essays written in Chinese, have explored the relationship between graph characteristics and essay quality systematically based on a scoring rubric. The proposed graph-based features measure spatial patterns of concepts implicated in an essay to capture the expressiveness of the main idea, the similarity with high-scoring essays’ content, and the distance between concepts to characterize the development of ideas. The purpose of this paper is not only to score Chinese essays but also to extend the construct coverage of automatic essay scoring for essays.

## Related Work

To predict the quality of an essay, researchers used a variety of computational linguistic features to capture the writing construct. The development of the AES field has been carried out in different research fields, as we will describe in the following sections.

### Computational Linguistic Features and Writing Quality

In recent years, the widespread use of online learning systems, word processing software, and text mining technology has accelerated the development of AES systems. The most widely adopted method or tool for computationally deriving linguistic features is based on natural language processing. One of the most commonly used text analysis tools is Coh-Metrix. The Coh-Metrix (Graesser et al., 2004), which was developed by Memphis University, is a web-based text analysis tool that was originally used to measure the textual coherence in American students’ reading textbooks. To this date, a variety of variables from the text can be extracted using Coh-Metrix, and these variables involve multiple aspects such as text cohesion and coherence, syntactic complexity, vocabulary information, and conceptual clarity. Nowadays, it is widely used in various fields of research and represents the current mature level of text mining, and has been proven to predict text quality (McNamara et al., 2015). In terms of the features currently used for automatic essay scoring, the feature values of AES mostly focus on some shallow language features. Language features such as grammar and syntax are the easiest to obtain and score. Taking e-rater as an example, nine of its eleven macro-features are language features, and the other two are content features. The nine language features are as follows: essay structure, writing, grammar, language use, mechanism, style, average word length, word frequency, and convention. Each language feature also contains a group of micro-features that are easy to count and calculate, which include spelling, punctuation, compound word rules, and so on. The feature of the structure depends largely on the length of the essay. In contrast, content features are more difficult to digitize, and we elaborate on this in section “Measuring the Ideas and Content of Essays.”

The Chinese-oriented AES study started relatively late compared with the English version. With the development of Chinese natural language processing technology (such as the maturity of Chinese word segmentation technology), a small number of Chinese AES research appeared in the early twenty-first century (Zhang and Ren, 2004; Li, 2006; Cao and Yang, 2007). Professor Liu and his team at Harbin Institute of Technology developed an automatic evaluation system for Chinese writing (Hao et al., 2014; Liu, 2015; Gong, 2016; Fu et al., 2018) and successfully implemented the scoring system on the national college entrance examination (NCEE), the Chinese proficiency test for minorities in China (MHK), and classroom writings. The features extracted by Chinese-oriented AES systems at first were mostly based on surface language features, such as word frequency (Zhang and Ren, 2004; Li, 2006). In recent years, some research has focused on the identification of rhetoric in Chinese essays, such as recognizing parallel and dual sentences (Gong, 2016) and identifying quotations, metaphors, and personifications (Liu, 2015). It is worth noting that researchers in other fields, such as computational linguistics, have also discovered some linguistic features related to the quality of Chinese essays. Zhang and Liu (2018) proposed a method for calculating the dependency distance based on the syntactic dependency treebank and found that Chinese writers have the largest dependency distance among the 20 languages studied. Wang and Liu (2019) research found that in Chinese writing, the syntactic dependence distance increases with the increase of a person’s Chinese writing ability, and linguists have found that the syntactic complexity of writing is related to a person’s writing ability. However, the contribution of syntactic complexity to the automatic evaluation of essays remains to be examined. This is why we consider dependency distance as an indicator of syntactic complexity and calculate a variable based on the dependency distance for predicting the quality of the composition.

This shows that current AES pays little attention to the characteristics of deep writing traits such as ideas and content when evaluating the quality of a composition. Therefore, this is exactly the controversial focus of AES. If the features cannot cover the main dimensions of the essay’s quality, the construction of an automatic scoring system is worthy of further discussion.

### Measuring the Ideas and Content of Essays

At present, the essay features that AES can measure and evaluate are mainly divided into two categories: one is the language style features, including vocabulary, grammar, language conventions, and mechanisms (such as English spelling and capitalization), the other is content or semantic features. The methods in the existing AES pay much attention to the evaluation of the first type of essay features. However, they can only evaluate the second type of content or semantic features to a certain extent. Meanwhile, most AES content evaluation methods adopt an approach that compares vocabulary and content of an essay with high-scoring essays to predict a content score (Landauer et al., 1998; Cao and Yang, 2007; Kakkonen et al., 2008; Attali, 2011; Li and Wen, 2017). The quality of ideas in essays is an important trait, but they are more difficult to measure than content, but some studies attempted this. Somasundaran et al. (2016) measured the development of ideas in essays, but not scoring the main idea. The main idea is also important in most rubric scoring, and is generally referred to as having a clear focus, and reflects the author’s position and perspective. Writing evaluation criteria or scoring rubric often regard the main idea as a crucial evaluation element. The main idea refers to the subjective feelings and thoughts expressed by the author (Cui, 2001). Because the AES developed by this research is used for the topical writing task, which is common in Chinese writing test, students need to review the prompt first and then determine how to express all parts of the essay. In recent years, Open Information Extraction, Semantic Networks, ontology, Fuzzy Logic, and Description Logic have been utilized in the design of AES systems (Zupanc and Bosnic, 2017), however, because the semantic granularity analysis is too difficult, it is tough to evaluate the quality of the content and main idea.

### The Use of Graphs in the Evaluation of Writing

Language is a symbolic system that is a mental model derived from perception and understanding (Garnham, 1981; Greeno, 1989; Johnson-Laird, 2005). Concept map graph structures can effectively assess the conceptualization of an essay by integrating the various ideas and concepts (Kim, 2013). As a structural knowledge representation consisting of concepts and relationships, the graph structure successfully represents an author’s thoughts (Seel, 1999, 2001; Zouaq et al., 2007; Zouaq et al., 2011; Villalon and Calvo, 2011). Linguists believe that the sentences in an essay include surface and deep structures, shape characteristics of the sentence, and semantic information about the deep structure (including the connections between concepts). The implicit meaning of the deep structure may be extracted by analysis of the surface structure (Bransford and Johnson, 1972; Fodor, 1974). Jin and Liu (2016) used modern Chinese as an example to study the structural features of human language as a multi-level system using a complex network method. Jin and Liu found that the four network models of Chinese (which are character co-occurrence, word co-occurrence, syntactic relationship, and semantic relationship) present their respective statistical characteristics and reflect the commonalities and connections on all levels of the system. The commonality and individuality of these systems indicate that there is a close relationship between language-related attributes and human cognition. In recent years, researchers have introduced the graph theory into the field of AES, and transform the text into a graph structure rather than vectors to explore the deep features of an essay (Somasundaran et al., 2016). The graph structure presents the development and semantic relationship between the words in an essay. Somasundaran used the characteristics of the graph structure obtained from an essay to capture the essay’s development. The research results show that the graph structure-based method can improve the scoring accuracy of the overall quality and the development of ideas. Ke et al. (2016) used complex networks to score Chinese essays and adopted in-/out-degrees, clustering coefficient, and dynamic network features. However, these studies are all based on lexical networks, the relationship between the features based on the graph, and the traits of the essay evaluation criteria was not explored systematically.

This study focused on two research questions:

1. What is the performance of graph-based features in the automatic scoring of essays?
2. Can the features based on a graph capture the quality of the main idea, content, and development of essays?

## Materials and Methods

**FIGURE 1:**
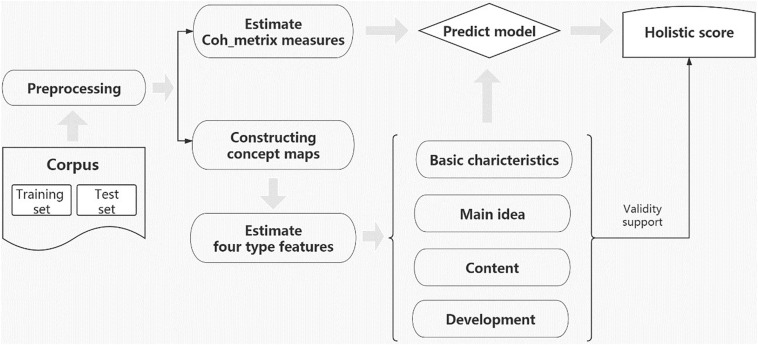
AECE architecture.

We introduce a graph-based approach for the Automated Evaluation of Chinese Essays (AECE) to automatically evaluate Chinese essays. This study establishes an automated model using essays from Chinese students and predicts their holistic scores. The modeling method was validated by examining the agreement between the AECE and human scores. The features used in this study are derived from graphs to capture the quality of ideas, content, and essay development. The process of how AECE works is shown in Figure 1.

### Data

The experiments were performed on a corpus made from a Mandarin Chinese language assessment for monitoring the teaching quality of basic education. Mandarin is the standard spoken and written Chinese language, and it currently has a penetration rate of about 70–80% in China. The essays were written by 15,000 grade eight middle school students from mainland China and constituted a total of six datasets. The male–female ratio of each dataset was between 7:9 and 8:9, and each student was only assigned to one prompt at random. Each dataset includes essays from one prompt. The number of essays within each dataset were 3,000, 3,000, 3,000, 2,000, 2,000, and 2,000, respectively. The six different prompts are as follows: Prompt 1: Company is the best gift; Prompt 2: If given another chance; Prompt 3: Something hard to forget; Prompt 4: Write a notification; Prompt 5: Write a proposal to save food; Prompt 6: A letter for a teacher. Each student is required to fully examine the prompt topic that was randomly assigned before writing and decide upon the main idea for the essay.

Because these essays come from a real test, all human raters have undergone rigorous training to master the scoring rubric (see Supplementary Appendix A). Each essay was scored holistically and for three traits: main idea, content, and development. For prompts 1, 2, and 3, each essay was simultaneously evaluated by two experts (human raters) to select any appropriate score from the integers 1 to 6 for scoring (with essay performance from poor (1) to excellent (6)). For prompts 4, 5, and 6, the scoring values are integers 1–4. If the scores from the two human raters differed by less than two points, the rounded-up mean of the two scores became the final score. A third rater scored the essay if the first two raters disagreed (the difference is greater than two points), and the final score comprised the mean of the two closest scores. Table 1 reports the information about the sample size and other characteristics of each dataset.

**TABLE 1:** The characteristics of the datasets from the six essay prompts.

	Prompt					
	1	2	3	4	5	6
Number of essays	3,000	3,000	3,000	2,000	2,000	2,000
Mean number of characters	433.29	426.89	414.62	158.06	128.88	154.20
SD of number of characters	153.67	150.12	137.56	47.24	47.94	51.36
Range of the rubric	0–6	0–6	0–6	0–4	0–4	0–4
Mean of human score	4.07	4.07	3.68	2.91	2.68	3.07
SD of human score	1.14	1.22	0.86	0.51	0.72	0.62

### Existing Linguistic and Discourse Features

We used 72 features as the baseline for this study to compare the performance of a feature set drawn from a graph. Most of the features were inspired by the Coh-Metrix and adapted to Chinese. We divided these features into seven groups to cover the different aspects of language discourse in an essay, and this includes basic description, lexical diversity, use of connectives, and essay coherence. All 72 linguistic features that we implemented in our baseline AES system (hereinafter referred to as “baseline features”) are presented and explained in Supplementary Appendix B. In particular, a group of LSA-based features, which measure the cohesion of essay, is included in the baseline features, and the LSA space was trained on all the original essays from the six prompts.

### Novel Features Based on Graphs

The features for measuring the quality of an essay are based on the assumption that the relation between concepts in a graph structure of a text can represent the meaning and ideas of the internal knowledge of the writer. To present a clear picture of the construction of graph-based features in this section, we will introduce the process of graph generation via an essay and the features extracted in detail.

#### Processing of the Graph Construction

##### Step 1: Preprocessing

In many languages, there is usually a space between words; however, there is no separation mark between Chinese characters except for punctuations. Thus, we have segmented the text to get words, which are the smallest independent units for expressing meaning. In this study, the period, question, and exclamation marks are regarded as the boundary of a sentence, and each text is divided into sentences. We used a Language Technology Platform Cloud (LTP)^2^ to obtain separated words (W_1_, W_2_, … W*_*m*_*) from each sentence. Furthermore, dependency syntax analysis and part-of-speech tagging are also performed using LTP in this step. Dependency syntactic analysis identifies the subject, predicate, adjuncts, and other sentence components and reveals the syntactic dependency relationship between the components, which is the basis for our later use of coreference resolution and establishing the connection between nodes.

##### Step 2: Identify concept as a node

In this study, we have distilled a set of concepts (C_1_, C_2_, … C*_*m*_*) that are as close as possible to the content of an essay from the word segmentation (W_1_, W_2_, … W*_*m*_*), so that each node in the graph can represent or be close to a *concept* rather than a *word*. Thus, four main tasks are included to push W → C. (1) Finding and filtering the stopwords that frequently appear in various topics and have little contribution to the ideas and content of the essay. A list of stopwords made up of 1,208 words was edited by researchers, including function words and some content words such as “want.” These words are determined in the pre-program optimization process and are ubiquitous in essays, but represent the content poorly. For example, “want” often leads to a higher weight and more connect edges, but lacks distinctive essay “concepts.” After a discussion with experts, a small number of these words were added to the stopwords list. (2) Coreference resolution. The purpose of coreference resolution is to associate the descriptive information about the same entity (such as representing a person, a location, or an organization) that might have been scattered in various sentences in a composition. The LTP (v1.0) program is used to detect coreferences in essays; whereas pronouns are replaced with the nouns they represent, except for first-person pronouns. (3) In addition to the stopwords in the text, there are also a large number of words that do not contribute much to the content of the essay, such as adverbs, numerals, prepositions, and spatial terms. We mainly retain nouns, first-person pronouns, verbs, adjectives, and related phrases, which are effectively and closely related to the ideas expressed, while filtering out other parts of speech. Our original intention was to refine the nodes to approximate a “concept” in the graph to reflect a student’s understanding of the writing task. Kim (2013) only used nouns or noun phrases as the nodes in the concept map of an essay. Here we add verbs, adjectives, and related phrases because they usually constitute the main components of sentences. Another reason is that the correlations between the frequency of these parts of speech and the essay score are greater than 0.40 (for example, noun: 0.56, verb: 0.57, adjective: 0.43), whereas other parts of speech are not. We pre-set the categories to be filtered in the program, and this process is automatically completed based on the results of the parts of speech sentence tagging. (4) Merge of synonyms. To extract the concepts in an essay, a key step is to merge words that have the same (or very similar) semantics using the Big Cilin^3^. The Big Cilin is a large-scale open-domain database of Chinese knowledge containing 10 million entities and more than 3.3 million relations. The entities in Big Cilin are divided into 12 categories, with 94 middle categories and 1,428 subcategories. Under the subcategories, the word groups are divided according to the synonymous principle, of which the finest level is the atomic word group. The words in each atomic word group share the same or very similar semantics. In this study, we take the word type that appears most frequently in the corpus as the *standard word type* to represent a synonym word group. It means, for example, we consider no difference between “computer” and “laptop” when evaluating a writer’s ideas. Thus, each distilled concept should be primarily stored as a standard word type, which we used later for calculating feature values. It is estimated that an average of 20–30 words are merged with synonyms based on Big Cilin in prompts 1, 2, and 3, whereas prompts 4, 5, and 6 averaged 5–10 words. In addition, because of the difficulty of multi-word concepts in natural language understanding and to reduce calculations and concentrate on the research, we have determined the meaning of polysemy under each prompt in advance. In the writing tasks for this study, the same prompt usually limits the content of a topic, so we assume that the concepts of polysemous words in the same prompt are the same, and we elaborate on this issue in the discussion section. Note that, in practice, all words and dependencies in an essay are used to construct the initial graph. Then the concepts are identified, and only valid concepts and related edges are retained as the final graph.

Finally, a set of concepts obtained through the aforementioned process serves as the node-set of the graph, and the number of nodes on a graph is considered as equal to the number of unique concepts in an essay. Through the processing of the essays in this study, the average number of nodes in the graph of prompt 1, prompt 2, and prompt 3 are about 20–30, and the average number of nodes in prompt 4, prompt 5, and prompt 6 are about 10–30 in general.

In addition, the weight of a node is calculated by dividing the number of occurrences of the word represented by the node in the essay by the total number of occurrences of all nodes in the graph.

##### Step 3: Construct edges

In the construction of relations between nodes, we adopt a method based on syntactic dependency to construct edges between concepts. We used LTP Dependency Syntax Analysis Module^4^ to parse the essays, and this is based on the universal dependency theory, but some amendments to Chinese natural language processing were made. The dependency parsing labeled attachment score of LTP achieves about 81–84%, and the unlabeled attachment score is about 83–86%. Some syntactic dependency relations are used for graph constructions. These relations between sentence components are relatively important for the organization of semantics in a sentence, which includes subject-predicate, modifier-head, apposition, combination, serial verb, and verb complement constructions. If there is one of these six dependencies between two nodes (or concepts), an edge is constructed between the two nodes. The weight of the edge is calculated by dividing the number of dependencies between the two connected nodes by the total number of dependencies between all nodes in an essay. The total number of dependencies is also calculated based on the six selected preferred dependencies. The process of converting an example essay into a graph can be found in Supplementary Appendix C. Hereinafter, all the words in the graph derived from an essay were translated into English in all figures.

#### Novel Features Based on Graphs

In this section, a novel feature set is computed based on an essay’s graph. It is assumed that the characteristics and structure of the graph will vary with the quality of the essay. To capture the node’s spatial patterns in the graph, we developed many features. Here, we introduce word2Vec to represent each node. Word2Vec is a well-known model used in natural language processing to represent words as a numeric vector. The word embeddings used in this study is the 300-dimensional word vector obtained by training the SVNG (skip-gram model with negative sampling) model based on the corpus of the Baidu encyclopedia^5^; see the details in Li et al. (2018). Then, we can calculate the semantic distance between two nodes based on the word similarities represented as word2Vec. It should be noted that the semantic distance between a pair of nodes is static, but the weight of the edge between the two nodes is dynamic because the weight depends on the dependencies found in each text. Then, four groups from the proposed features are calculated.

##### Basic measures of graphs

Four indicators, which are the numbers of nodes, number of edges, the average of the node degree, and syntactic complexity derived from the graph, are used to measure the complexity of the vocabulary (concept) used in an essay.

###### Number of nodes (NN).

It refers to the number of nodes in the graph; it indicates the types of words used and the complexity of the essay. We used this indicator based on the following two considerations: First, it is a description of the concept rather than the word, so it is less likely to depend on the essay’s length. Essay length and the score is highly correlated in general, but it measures higher-order writing skills inadequately (Attali, 2015). Second, it is related to the complexity of the ideas in an essay, which is useful for explaining the quality of the ideas.

###### Number of edges (NE)

It refers to the number of edges in a graph, and it represents the relationship among the concepts on the graph. The greater the number of edges in the graph, the more the concepts are connected to each other in the essay.

##### Average degree (AD)

The degree of a node refers to the number of edges associated with the node. The higher the degree, the more important the node is in the essay (Ke et al., 2016). In a graph constructed based on syntactic relationships, each node has an average syntactic relationship with three to five other nodes. A higher degree means that there is more syntactic relationship with this node and other nodes. When the lengths of the two essays are basically the same, the higher the average degree means there are more connections between the concepts in the essay. The average degree is calculated as Eq. 1:

(1) $$A\,D=\left(1/N\right)\,\sum_{i=1}^{N}w_{v_{i}}\,D\,e\,g\,r\,e\,e_{i}$$

where *N* is the number of nodes in the graph and *Degree*_*i*_ is the number of edges associated with the node *i*. *w*_*v*_*i*__ is the weights of node *i* in the graph.

###### Syntactic complexity (SC)

The “dependency distance” refers to the linear distance between one word as the governor and another word as the dependent in a sentence (Heringer et al., 1980), and is generally measured by the number of words in the middle interval (Hudson, 1995). In the dependency syntax processing model, this distance is closely related to human cognitive load; therefore, dependency distance is also often regarded as an important indicator of syntactic complexity (Liu, 2008). In a graph based on syntactic relationships, the dependency distance between any pair of concepts with syntactic relationships is defined as the sum of the dependency distances divided by the weight of their edges in the graph. To clarify, the nodes in the graph from the original text are collapsing over multiple instances in the essay, so the node pairs in the graph may have more than one syntactic relation between them. The average dependency distance of the concepts in an essay reflects the syntactic complexity of the essay and is calculated as Eq. 2:

(2) $$S\,C=\left(1/n_{s\,y\,n}\right)\,\sum_{k=1}^{n_{s\,y\,n}}\left[s\,u\,m\,\left(D_{i\,j}^{k}\right)/w_{i\,j}^{k}\right]$$

where *n*_*syn*_ refers to the number of concept pairs with syntactic relationships, *k* represents the *k*th syntactic pair in the graph, $D_{i\,j}^{k}$ is the sum of the syntactic dependency distances between the concepts *i* and *j* with syntactic relationships, and $w_{i\,j}^{k}$ represents the weight of the corresponding edges of concepts *i* and *j* in the graph.

##### Measures of the main idea

One of the most distinct characteristics of an excellent essay is a clear main idea. Previous studies have stated that the global aggregation graph measurement can predict the cohesion of the semantics in an essay (Zupanc and Bosnic, 2017). This study assumes that the stronger the global aggregation of the graph structure is, the more prominent the main idea of the essay will be. Therefore, three features of global aggregation were extracted to measure the essays’ main idea.

###### Mean of PageRank value (MPR)

PageRank (Brin and Page, 1998) is an algorithm used to rank the importance of a node in a network. In a graph, the importance ranking of a node is the sum of the weighted rankings of all nodes connected to it, and it simulates a “random walk” on a graph. For a node, if more nodes are connected to it, or the connecting node has a higher PageRank value, then that node will have a higher probability of random accessing that, and greater importance. The PageRank value calculation depends on the network topology; once the network topology (a connection) is confirmed, PageRank value is determined, and it is usually an iterative process; the maximum number of iterations is 100 in the present research. In an essay’s graph, the more chances that a word (or concept) is visited (or connected), the greater its influence. Furthermore, the word connected to it also has influence. Then, the higher the MPR value signifies that there are more probabilities of a high connectivity concept.

###### Moran’s I (MI)

Moran’s I (MI) is a classic spatial autocorrelation measurement that expresses the global clustering situation of points in space. If the values of variables in space become more similar to the reduction of distance, it means that the data are clustered in space. This is referred to as a positive spatial correlation. If a text shows positive spatial autocorrelation, it indicates that the parts of the text are well related to each other (Kim, 2013). On the contrary, if the measured value grows with the reduction of distance, the data are scattered in space and is referred to as a negative spatial correlation. This indicates the text has a lack of dependence and contains randomness. In this study, the graph exists in a high-dimensional semantic space based on word2Vec, and the coordinates of each node represent its semantic, which is fixed in the space; whereas the grammatical relationship between nodes defines whether a pair of nodes are connected, and their relationship should differ due to different essays. Furthermore, the MI value reflects the degree of semantic aggregation between adjacent nodes. This implies that the larger the value, the semantically related words (or concepts) in the essay will cluster together. To suit the 300-dimensional word2Vec representation for a node in this study, Zupanc and Bosnic (2017) method was adopted to calculate this measurement. The adjustment from the original two-dimensional space is as follows:

(3) $$Moran^{\prime \prime }sI=N/S\cdot n\sum_{k=1}^{n}[\sum_{i=1}^{N}\sum_{j=1}^{N}w_{i\,j}\left(D_{i}^{k}-\bar{D_{c}^{k}}\right)\left(D_{j}^{k}-\bar{D_{c}^{k}}\right)/\sum_{i=1}^{N}{\left(D_{i}^{k}-\bar{D_{c}^{k}}\right)}^{2}]$$

where *N* is the number of points (or concepts) in a graph and *n* is the number of dimensions on the word2Vec representation. So,$\,n=300;\,D_{i}^{k}$ *k* = 1 … *n*; *i=1*, …, and *N* is a *k*th dimension of concept *i*; $\bar{D_{c}^{k}}$ is a *k*th dimension of a mean center. Weights (*w*_*ij*_) are assigned to every pair of concepts, with the value *w*_*ij*_ = 1. If *i* and *j* are neighbors, then it means there is an edge between *i* and *j*; otherwise, the value *w*_*ij*_ = 0 and *S* are a sum of *w*_*ij*_. To avoid negative values, we normalized this measure before using it.

##### Similarity with high-score essays (SHSE)

To measure the consistency of an essays’ content in relation to the prompt topic, this study uses the similarity of graphs to approximate the similarity of an essay. It is based on the assumption that the graphs of two similar essays are also similar. The closer the graph of an essay is to the high-scoring essay, the more its content adheres to the prompt. This method is more common in the field of AES, that is, each essay (target essay) is compared with the high-scoring essays or the standard text responses to compute the similarity between the target essay and the high-scoring essays based on different natural language processing approaches, such as the CVA (Attali, 2011), the LSA or extended LSA method (Landauer et al., 1998; Cao and Yang, 2007; Mikolov et al., 2013), LDA method (Kakkonen et al., 2008), and so on.

In this study, high-scoring essays refer to the highest-scoring essays written by the students for each prompt in the training set (i.e., the essays scored 6 in prompt 1, prompt 2, and prompt 3, and the essays scored 4 in prompt 4, prompt 5, and prompt 6). Each prompt gets a subset of high-scoring essays, and each target essay in a prompt is compared with the same high-scoring essays of this prompt to calculate the similarity between the target essay and the high-scoring compositions. Then the information of the nodes, edges, and weights in a graph drawn from the target essay and the graph drawn from the high-scoring essay set is used to calculate the similarity to the high-score essay. The formula is as Eq.  4:

(4) $$\begin{array}{l} Similarity(CM_{i},CM_{H})\\ =\text{α}\left(\sum^{\text{​}}\forall Common\;node(\min({\text{w}}_{{\text{v}}_{\text{i}}},{\text{w}}_{{\text{v}}_{\text{H}}})/\max({\text{w}}_{{\text{v}}_{\text{i}}},{\text{w}}_{{\text{v}}_{\text{H}}}))\right)\\ /max(m_{CM_{i}},m_{CM_{H}})+\\ (1-\text{α})\left(\sum^{\text{​}}\forall Common\;edge(\min(w_{e_{i}},w_{e_{H}}\right)\\ /\max(w_{e_{i}},w_{e_{H}})))/max(n_{CM_{i}},n_{CM_{H}}) \end{array}$$

where the similarity between a graph (*CM*_*i*_) and a graph of a high-scoring essay set (*CM*_*H*_) is noted as Similarity(*CM*_*i*_,*CM*_*H*_), which is taken as the similarity between an essay(*e*_*i*_) and high-scoring essays. *m*_*CM*_*i*__,*m*_*CM*_*H*__ are the number of the nodes in *CM*_*i*_ and *CM*_*H*_, respectively, *n*_*CM*_*i*__,*n*_*CM*_*H*__ are the number of the edges in *CM*_*i*_ and *CM*_*H*_; max(*m*_*CM*_*i*__,*m*_*CM*_*H*__) are the maximum number of nodes in *CM*_*i*_ and *CM*_*H*_; max(*n*_*CM*_*i*__,*n*_*CM*_*H*__) are the maximum number of edges in *CM*_*i*_ and *CM*_*H*_. *w*_*v*_*i*__ and *w*_*v*_*H*__ are the weights of the identical nodes in *CM*_*i*_ and *CM*_*H*_, respectively, *w*_*e*_*i*__ and *w*_*e*_*H*__ are the weights of the identical edges in *CM*_*i*_ and *CM*_*H*_. α ∈ (0,1) is taken as 0.5 in this study.

The first half of Eq. 5 indicates the similarity between the nodes in the graph *CM*_*i*_ and *CM*_*H*_. When they have more of the same nodes between them, the greater the similarity between the two graphs. Considering that the importance of nodes contributes differently to ideas in different essays, we use the frequency percentage of a concept in an essay as the weight of the node. The second half of Eq. 5 is a measure of the similarity of the edges in the concept graph. For the identical edges in *CM*_*i*_ and *CM*_*H*_, the closer *min*⁡(*w*_*e*_*i*__, *w*_*e*_*H*__)/*max*⁡(*w*_*e*_*i*__, *w*_*e*_*H*__) is to 1, the more the number of identical edges there are, and the greater the similarity between the two graphs.

##### Essay development

The development of a concept reflects the compactness and development of an author’s thoughts. The edges on the graph reflect the ideas development path. The closer the semantic distance between adjacent concepts are, the higher the consistency of the development of ideas.

###### Weighted average of the distance between two adjacent points (WDAP)

The distance between the two points reflects the semantic distance between two adjacent concepts. The neighboring points refer to the pair of points with an edge between them. To calculate it, the sum of all the Euclidean distances are measured based on word2Vec between two neighboring concepts and then dividing it by the sum of the weighted edges.

###### Weighted average distance to the nearest neighbor points (WDNP)

This metric measures the average of the minimum Euclidean distance between neighboring points. If there is more than one neighbor on a node, and there is an edge between each neighbor and this node, then the one closest to this node is its nearest neighbor.

### Model Building and Scoring

To construct the prediction model for holistic human scoring, each dataset of human-scored essays was randomly divided into two sets: training and test. The training set consisted of 66.7% of the total sample, with the test set containing 33.3%. The training set was used to develop the scoring model, and the test set was utilized to predict the scores and evaluate the prediction accuracy. Table 2 indicates the characteristics of the training and test sets. A multiple linear regression (MLP) model was adopted in this study mainly because it is necessary to assess the relative weight of the features when scoring different aspects of an essay. To build a scoring model, the values from the training set are used to determine the appreciate weight for each feature. To evaluate the model, the values of the extracted features from the test set are multiplied by the weights generated from the training set to predict the holistic scores of the essay. The quadratic weighted Kappa (QWK) and exact agreement (the rounded scores are exactly in the same point) are used to evaluate the accuracy of predicting the human scores on the test set.

**TABLE 2:** Description of the training and test sets.

		Prompt					
		1	2	3	4	5	6
Training set	Number of essays	2,100	2,100	2,100	1,400	1,400	1,400
	Mean number of characters	433.60	426.20	414.10	158.47	129.00	154.36
	SD of number of characters	153.38	150.22	139.20	47.03	47.35	51.11
	Range of the rubric	0–6	0–6	0–6	0–4	0–4	0–4
	Mean of human score (H1)	4.06	4.08	3.67	2.91	2.69	3.07
	SD of human score (H1)	1.15	1.22	0.86	0.50	0.70	0.60
Test set	Number of essays	900	900	900	600	600	600
	Mean number of characters	432.58	428.50	415.84	157.11	128.61	153.84
	SD of number of characters	154.42	149.97	133.74	47.75	49.34	51.97
	Mean human score (H1)	4.08	4.05	3.70	2.90	2.65	3.08
	SD of human score (H1)	1.12	1.21	0.86	0.52	0.77	0.65

*SD = standard deviation*

## Results

The performance of the model is evaluated based on the six data sets. First, the automatic score and human scores were compared in terms of agreement (QWK and Exact agreement) among the raters. Second, we started with the descriptive statistics of the human score and the individual feature used in the model, and then we analyzed the correlation between the feature value and the human score and determined the relative importance of each feature. At last, the results of the assessment accuracy of the subgroup were analyzed.

### The Performance of AECE

In this study, model training was based on the first manual score (H1), and model testing was based on comparing the automatic score with the second human score (H2). Table 3 lists the Kappa values of the models and the exact agreement values for all the prompts. The first column shows the results (QWK or exact agreement) between the holistic scores of H1 and H2, and the subsequent columns show the results between the automatic holistic score of each prompt and the H2 holistic score. AECE is built on all the features based on a graph, and it includes the number of nodes (NN), number of edges (NE), average degree (AD), syntactic complexity (SC), mean of PageRank value (MPR), Moran’s *I* (MI), the weighted average of the distance between two adjacent points (WDAP), weighted average distance to the nearest neighbor points (WDNP), and similarity with high-score essays (SHSE).

**TABLE 3:** QWK and exact agreement of human raters (H1/H2) and H2/AECE holistic score, p values are computed for QWK.

		Comparison with H2					
		Prompt 1	Prompt 2	Prompt 3	Prompt 4	Prompt 5	Prompt 6
H1	QWK Exact agg.	0.72	0.73	0.69	0.74	0.73	0.77
		0.64	0.60	0.61	0.73	0.72	0.75
Baseline	QWK	0.77	0.78	0.70	0.78	0.76	0.79
	Exact agg.	0.67	0.66	0.63	0.78	0.74	0.79
AECE	QWK	0.86	0.81	0.79	0.88	0.87	0.89
	Exact agg.	0.71	0.68	0.61	0.84	0.81	0.88
Baseline + AECE	QWK	0.88	0.84	0.81	0.91	0.91	0.93
	Exact agg.	0.73	0.70	0.63	0.89	0.83	0.90
*p-*value	AECE/baseline	0.00*	0.00*	0.00*	0.00*	0.00*	0.00*

*p < 0.05.

The performance of AECE to predict the holistic score is statistically significantly different from the six data sets baseline. The QWK of AECE is not lower than or even 8–14 percentage points better than the human score. Although the baseline is better than the human agreement (1–5 percentage), the model’s QWK can be improved by 3–11 percentage points when the concept graph feature is added to the baseline feature (baseline + graph). Except for prompt 3, the QWK is above 0.80. The QWK difference between prompt 3 and the other prompts is relatively smaller when comparing the baseline and manual scores. Because the last three prompts have only 4 point intervals, the model accuracy on the last three prompts is higher than that of the first three. The QWK of AECE ranges from 0.79 to 0.86 on longer essays (prompt 1, prompt 2, and prompt 3) and ranges from 0.87 to 0.89 on shorter essays (prompt 4, prompt 5, and prompt 6).

All features are also used to predict the score of a trait in an essay. Table 4 reports the results of a feature set that scores the main idea, content, and essay development. The three types of feature sets evaluate the main idea, content, and essay development separately. The main idea feature set has the best predictive performance (QWK∈[0.70, 0.83]); the content feature has the second-best performance (QWK∈[0.65, 0.78]), and the weakest predictive performance is the developmental feature set (QWK∈[0.63, 0.75]). Each feature set performs better than the baseline model on its own target traits. This result indicates that graph features capture writing constructs that were not captured by the baseline feature set.

**TABLE 4:** QWK of feature sets on trait scoring (main idea, content, and development) on the six data sets.

Trait	Feature set	Comparison with H2					
		Prompt 1	Prompt 2	Prompt 3	Prompt 4	Prompt 5	Prompt 6
Main idea	H1	0.62	0.59	0.57	0.65	0.64	0.69
	Baseline	0.69	0.64	0.61	0.62	0.60	0.66
	Main idea	0.78*	0.72*	0.70*	0.81*	0.79*	0.83*
	Baseline + AECE	0.83*	0.74*	0.73*	0.87*	0.82*	0.87*
Content	H1	0.64	0.61	0.60	0.66	0.67	0.70
	Baseline features	0.60	0.55	0.61	0.69	0.66	0.72
	Content feature	0.72*	0.69*	0.65*	0.75*	0.73*	0.78*
	Baseline + AECE	0.76*	0.74*	0.69*	0.81*	0.76*	0.82*
Development	H1	0.55	0.52	0.50	0.58	0.53	0.61
	Baseline	0.61	0.56	0.58	0.67	0.65	0.69
	Development	0.67*	0.64*	0.63*	0.73*	0.71*	0.75*
	Baseline + AECE	0.71*	0.67*	0.68*	0.78*	0.74*	0.81*

The main idea features: MPR and MI. The content feature: the similarity with high-score essays. The development features: WDAP and WDNP. *p < 0.05, compared with baseline features.

### Analyses of the Performance of Features

Table 5 presents the mean, SD, and skewness scores for each feature. Most of the features have relatively small skewness values, and correlation analysis is used to study the relationship between all the graph-based feature and the different points of the essay. Table 6 contains six data sets and presents the average correlation (Pearson’s r) between each feature and the different scores from the second human scorer. Except for feature SC, the correlations between all the features and the holistic score are greater than 0.30 (*p* < 0.005, significant; results in the tables are omitted). The correlation between the values of a feature built for a particular trait and that trait score is between 0.50 and 0.78.

**TABLE 5:** Descriptive statistics of the features.

Prompt		NN	ND	AD	SC	MPR	MI	WDAP	WDNP	SHSE
1	Mean	30.15	123.59	12.23	1.68	4.07	0.61	9.18	7.93	0.68
	STD	4.73	15.54	3.17	0.05	0.19	0.10	1.02	1.38	0.07
	Skewness	–0.89	–0.34	–0.78	0.04	0.19	0.02	0.23	0.16	0.11
2	Mean	28.72	107.29	11.49	1.59	4.01	0.59	9.07	7.89	0.61
	STD	4.65	13.64	2.83	0.06	0.13	0.07	1.05	1.24	0.08
	Skewness	–0.57	–0.30	–0.53	0.03	0.12	0.03	0.12	0.17	0.13
3	Mean	24.61	103.73	11.02	1.65	3.94	0.60	9.01	7.86	0.60
	STD	4.31	14.21	2.91	0.03	0.21	0.08	1.04	1.21	0.05
	Skewness	–0.74	–0.19	–0.77	0.02	0.13	0.04	0.19	0.23	0.09
4	Mean	16.54	61.87	11.98	1.21	3.15	0.58	8.78	7.47	0.71
	STD	4.64	5.43	1.08	0.07	0.11	0.09	0.96	1.22	0.04
	Skewness	–0.33	–0.13	–0.72	0.07	0.09	0.03	0.17	0.14	0.08
5	Mean	13.87	56.26	10.81	1.15	2.97	0.51	8.69	7.30	0.69
	STD	3.19	4.03	0.07	0.03	0.08	0.05	0.68	1.11	0.03
	Skewness	–0.25	–0.17	–0.63	0.02	0.06	0.01	0.10	0.14	0.05
6	Mean	16.32	59.53	11.43	1.36	3.03	0.55	8.72	7.35	0.70
	STD	4.07	5.36	0.09	0.08	0.10	0.06	0.92	1.19	0.06
	Skewness	–0.35	–0.25	–0.56	0.04	0.07	0.02	0.13	0.16	0.03

**TABLE 6:** Average correlations (across all prompts) of feature values with H2.

Feature	Holistic	Main idea	Content	Development
NN	0.49	0.40	0.43	0.38
ND	0.43	0.27	0.35	0.27
AD	0.31	0.25	0.24	0.21
SC	0.23	0.14	0.20	0.18
MPR	0.68	0.78	0.52	0.42
MI	0.31	0.54	0.46	0.24
WDAP	0.39	0.23	0.25	0.63
WDNP	0.38	0.20	0.24	0.51
SHSE	0.55	0.48	0.64	0.47

**FIGURE 2:**
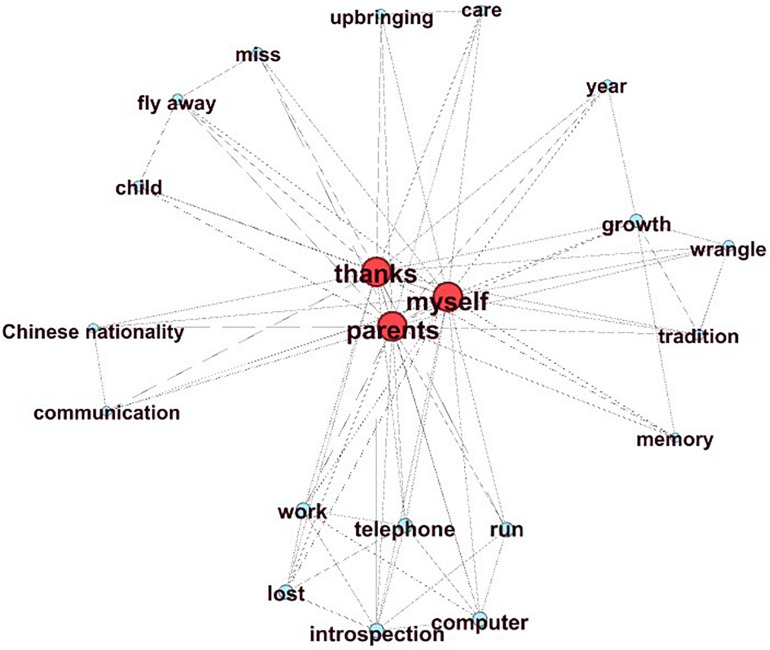
High-level main idea.

**FIGURE 3:**
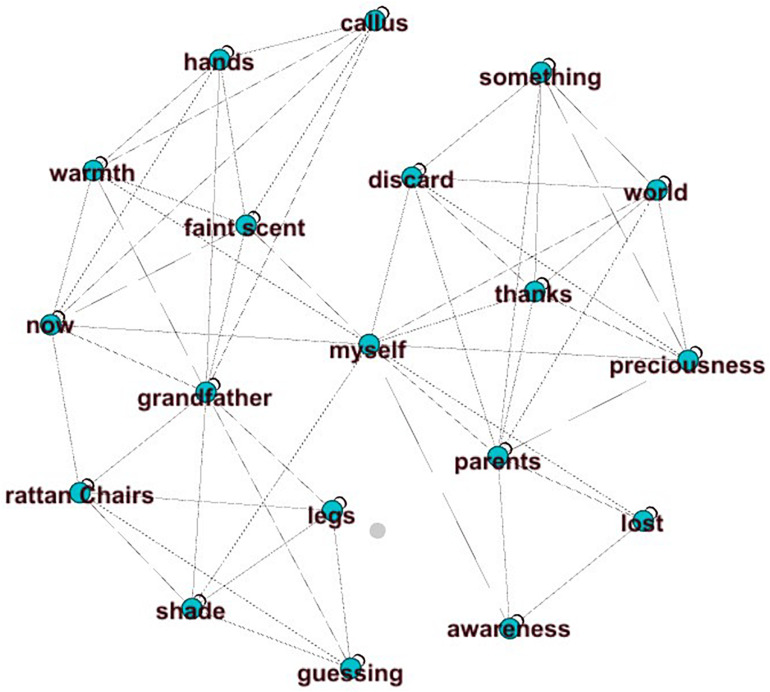
Low-level main idea.

MPR and MI have a high correlation with the main idea score, which means that the higher the value of MPR and MI, the better the essay’s main idea. Figure 2 shows a graph of an essay with a high main idea score (main idea score = 6), and Figure 3 shows a graph with a low main idea score (main idea score = 2). The higher the global aggregation of the graph, the more it tends to present a clear center. The center of the graph shown in Figure 2 may be “I am grateful to my parents.” This main idea is supported by the concepts in the other parts of the network (MI value = 0.83, MPR value = 4.97). In contrast, Figure 3 does not show a clear center, so the connections of all the nodes are evenly distributed, and the MI and MPR values are very low (MI value = 0.14, MPR value = 2.07).

**FIGURE 4:**
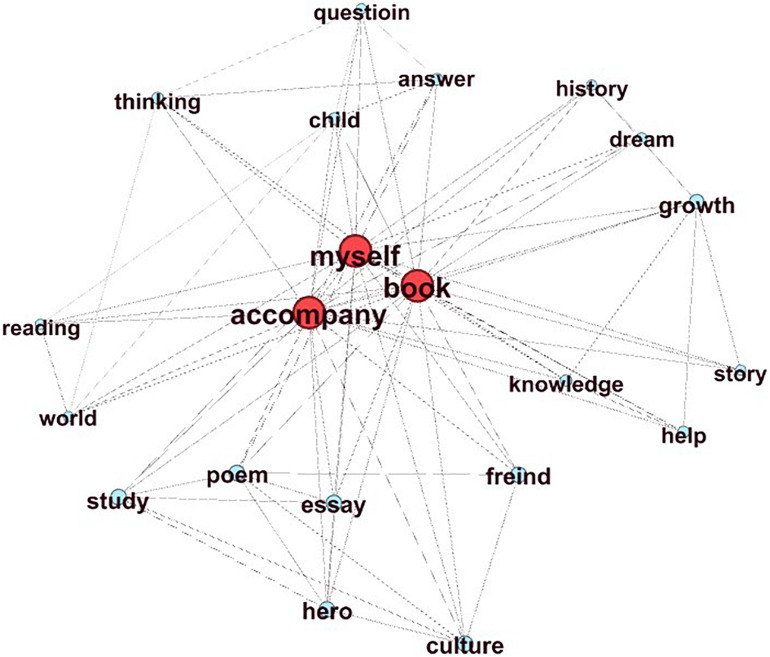
High-level content.

**FIGURE 5:**
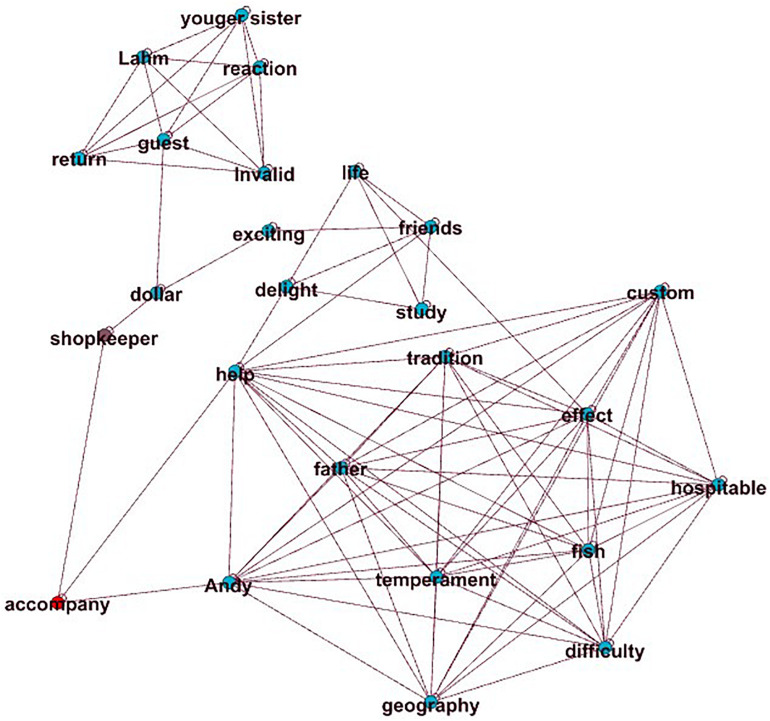
Low-level content.

The correlation coefficient between SHSE and the content score is the highest (*r* = 0.64) among all features. It calculates the similarity between the essay and the high-scoring essay on the same prompt. Here, the implicit assumption is that two similar essays transform two graphs with similar concept organizations. Figures 4, 5 show an essay with a high-content score (content score = 6) and an essay with a low content score (content score = 1) (Figure 5) from the same prompt (Prompt 1: Companionship is the best gift). Because the graph of the high-scoring essay is large, we chose one of the high-scoring essays to illustrate the difference. Figure 4 not only has a clear central “parent companionship” but also develops around the core concept of “accompaniment” (SHSE value = 0.91). Figure 5 shows an “off-topic” essay that uses concepts that rarely appear in high-scoring essays. The main body of its graph completely deviates from the key node “companion” and has little connection with it. In addition, the SHSE in Figure 5 is small (SHSE value = 0.28), which shows that the concepts have few intersections with the concepts of the high-scoring essay set.

**FIGURE 6:**
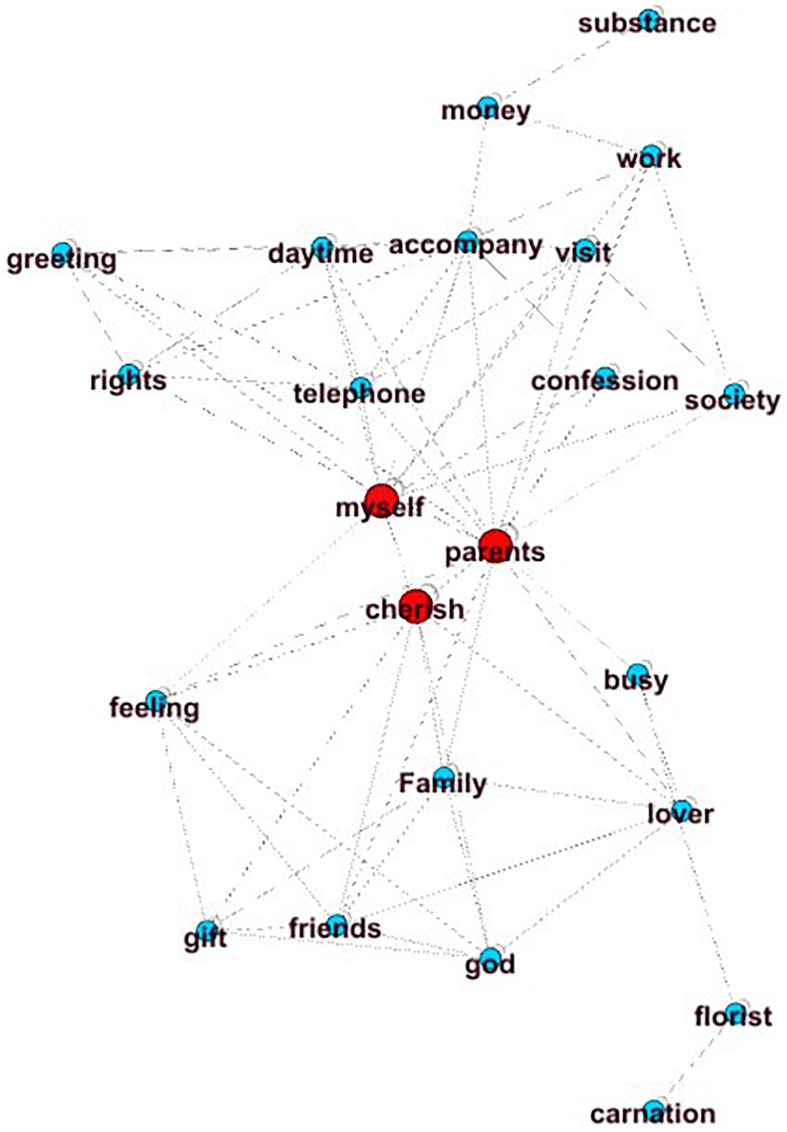
High-level development.

**FIGURE 7:**
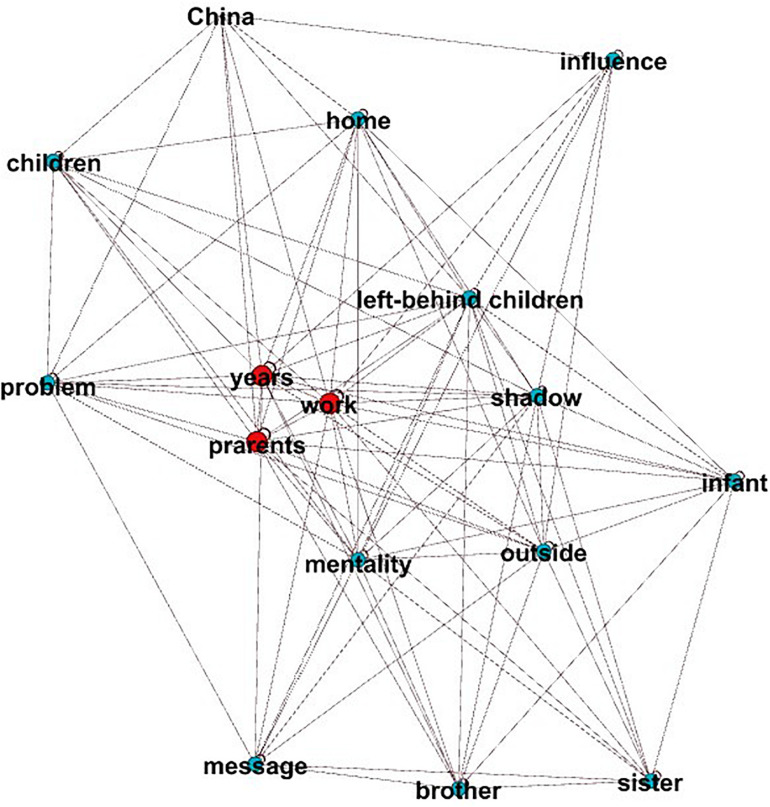
Low-level development.

The correlation between the values of WDAP and WDNP and the development score is high in the feature set, and the correlation coefficients between the values of the other features and the development score are lower than those of other traits. The smaller the value of these two features, the higher the average semantic similarity of the concepts used in the essay, so as to the less developed the content of the essay. Figures 6, 7 compare two graphs between an essay with a high development score and a low development score. The essay used in Figure 6 is fully developed, and it contains rich concepts and relatively long paths connected by edges, and it has above average WDAP (9.97) and WDNP (8.94). Compared with Figure 6, the concepts in Figure 7 are not rich enough, and the distance between the concepts is shorter (WDAP = 7.84, WDNP = 6.23), which shows that the essay is not fully developed.

To examine the relative importance of all features in predicting the holistic and trait score of an essay in the regression model of AECE, Table 7 gives the average relative weights of each feature in AECE across six prompts. The last column shows the coefficient of variation of the weight value between each prompt. This coefficient is calculated as the ratio of the SD to the mean and is expressed as a percentage. Obviously, the weight of the main idea is the largest in the total score prediction, which is understandable, because whether there is a prominent main idea or not is the most important scoring requirement for an essay, and a graph is better at capturing the global distribution of information in an essay. The relative weights of the developmental features are the lowest in the three types of features. The table shows that the weight of the features on different topics fluctuates relatively little, and most of the coefficients are between 20 and 45%.

**TABLE 7:** The relative importance of features (expressed as percent of total weights) from regression for the prediction of H1.

Features	Holistic		Main idea		Content		Development	
	Average	Coef. Var.	Average	Coef. Var.	Average	Coef. Var.	Average	Coef. Var.
NN	13	64	6	47	14	54	10	39
ND	9	39	5	67	8	34	8	54
AD	8	21	5	44	6	20	9	42
SC	7	26	5	29	4	32	5	31
MPR	26	20	37	23	21	36	13	25
MI	8	23	15	32	6	61	5	39
WDAP	7	31	8	25	6	25	18	27
WDNP	6	28	7	22	4	47	17	23
SHSE	16	47	12	36	31	24	15	35

### Fairness to Different Subgroups

When using automatic scoring instead of human scoring, the model accuracy of different subgroups should be assessed. We examined the subgroup performance of the model on different genders and different economic development areas. Standardized mean difference (SMD) was applied to evaluate the variability of AES in subgroups. If the variance exceeded the mean of 0.10 SDs, it would be flagged as a concern for the subgroups and require further evaluation (Bridgeman et al., 2012). Although the SMD of AECE exceeds the flagging criterion of 0.10 in absolute value in some subgroups, the prediction accuracy of AECE is higher than the baseline, and Table 8 shows the SMD of AECE is smaller than that of the baseline in each subgroup. Therefore, it can be expected that AECE will not introduce any substantial unfairness to the scoring process.

**TABLE 8:** SMD of AECE for different genders and economic development areas.

Prompt	Gender	Holistic		Main idea		Content		Development	
		Baseline	AECE	Baseline	AECE	Baseline	AECE	Baseline	AECE
1	Male	−0.13	−0.05	−0.09	−0.04	0.19	0.12	–0.27	–0.18
	Female	−0.11	−0.04	−0.14	−0.09	0.24	0.11	–0.19	–0.07
2	Male	−0.16	−0.11	−0.18	−0.07	0.23	0.15	–0.31	–0.19
	Female	−0.15	−0.08	−0.14	−0.10	0.19	0.13	–0.25	–0.09
3	Male	−0.14	−0.08	−0.12	−0.05	0.25	0.14	–0.28	–0.22
	Female	−0.12	−0.07	−0.15	−0.08	0.29	0.13	–0.20	–0.20
4	Male	−0.03	−0.01	−0.07	−0.03	0.08	0.04	0.21	0.10
	Female	−0.02	−0.01	−0.05	−0.01	0.09	0.02	0.13	0.09
5	Male	−0.06	−0.04	−0.09	−0.05	0.14	0.06	0.25	0.15
	Female	−0.05	−0.03	−0.07	−0.04	0.13	0.05	0.19	0.14
6	Male	−0.04	−0.02	−0.08	−0.03	0.09	0.04	0.15	0.12
	Female	−0.02	−0.02	−0.07	−0.03	0.07	0.04	0.14	0.11

**Prompt**	**Area**	**Holistic**		**Main idea**		**Content**		**Development**	
		**Baseline**	**AECE**	**Baseline**	**AECE**	**Baseline**	**AECE**	**Baseline**	**AECE**

1	Developed	−0.15	−0.09	−0.17	−0.09	0.19	0.13	–0.23	–0.14
	Developing	−0.14	−0.08	−0.14	−0.05	0.14	0.09	–0.17	–0.13
	Underdeveloped	−0.17	−0.10	−0.21	−0.08	0.15	0.17	–0.19	–0.15
2	Developed	−0.17	−0.12	−0.19	−0.12	0.23	0.14	–0.25	–0.17
	Developing	−0.16	−0.09	−0.16	−0.10	0.19	0.10	–0.20	–0.15
	Underdeveloped	-0.19	−0.13	−0.23	−0.14	0.27	0.19	–0.27	–0.18
3	Developed	−0.16	−0.10	−0.20	−0.13	0.20	0.14	–0.26	–0.16
	Developing	−0.14	−0.07	−0.17	−0.09	0.16	0.10	–0.21	–0.14
	Underdeveloped	−0.18	−0.12	−0.21	−0.16	0.18	0.13	–0.28	–0.19
4	Developed	−0.09	−0.02	−.13	−0.04	0.13	0.04	–0.12	–0.09
	Developing	−0.04	−0.01	−0.11	−0.03	0.14	0.02	–0.13	–0.07
	Underdeveloped	−0.11	−0.04	−0.16	−0.09	0.17	0.06	–0.21	–0.13
5	Developed	−0.12	−0.07	−0.17	−0.05	0.18	0.07	–0.17	–0.12
	Developing	−0.10	−0.03	−0.15	−0.02	0.14	0.05	–0.15	–0.10
	Underdeveloped	−0.15	−0.09	−0.19	−0.09	0.19	0.09	–0.29	–0.15
6	Developed	−0.11	−0.03	−0.14	−0.05	0.15	0.05	–0.16	–0.10
	Developing	−0.08	−0.02	−0.10	−0.02	0.12	0.02	–0.14	–0.08
	Underdeveloped	−0.13	−0.05	-0.15	−0.06	0.16	0.08	–0.18	–0.13

## Discussion

Overall, the findings in this study support a basic assumption that the quality of an essay can be captured by graph characteristics. The multi-faceted graph features show a better performance in predicting holistic essay scores and grasps the scoring construct of writing better than baseline features.

Previous studies based on language networks for essay scoring gave us an opportunity to connect text quality to graph characteristics. The main difference between this research and previous research lies in three distinct variables: (1) constructing a network based on “concepts” and “syntactic relations” instead of “words” and “co-occurrence relations,” thereby removing noisy information not related to the ideas; (2) linking the essay’s scoring rubric with the statistics of the network characteristics allows us to have an insight for what aspects of the essay’s qualities are measured; (3) some features in AECE combine graph characteristics with word embeddings to achieve fusion of global pattern of nodes and local semantic information of concepts. We will elaborate on these three issues in the following section.

First, a graph can reflect the global distribution of the organizational structure of the concepts used by the author in a writing task. In this study, the Big Cilin was used to merge the synonyms. Unlike previous studies (Ke et al., 2016; Somasundaran et al., 2016; Zupanc and Bosnic, 2017), a set of concepts in an essay were extracted instead of the original words due to the fact that there is no concept recognition and/or synonym merging, and the central node in the network is likely to be a preposition (Poiret and Liu, 2019) that has little contribution to the essay’s ideas. Please note that a systematic evaluation of synonym-merging is not presented in this paper. If these words enter the network, the center of the graph may deviate from the main idea of the essay, and the features (MI, MPR) that measure the aggregation of the network will no longer reflect the aggregation of the ideas. Table 6 shows that the number of concepts has a statistically positive correlation with the essay’s holistic and trait scores. Table 7 shows that the number of concepts contributed 13% to the essay’s predicted holistic score, 6% to the main idea, 14% to the content, and 10% to the coherence. In addition, from the perspective of the graph structure, the edges of the concept graph in this study have syntactic relationships between concepts, which reveal more information about the logical language relationship than the simple co-occurrence relationship (Ke et al., 2016; Somasundaran et al., 2016) used in previous research. Table 6 shows that the number of edges based on the grammatical relationship is also positively correlated with the essay score, and the predicted contribution range of the score is 5–9%. In addition, it seems that the more concepts and the richer the relationship between the concepts, the higher the essay’s quality. This is in line with our expected writing ability due to common sense.

Second, in previous studies, the relationship between graph-based features and the essay scoring rubric has not been analyzed. The meaning of these features in essay scoring is ambiguous so as to reduce the validity of the scoring model (Powers et al., 2002). This study constructs features from three aspects: main idea, content, and development. These features are related to the scoring rubrics used in this research. This study draws on previous research results from computational linguistics and natural language processing methods and builds a predicted model based on real writing test data. Table 3 shows that these graph-based scoring features show promising performance in the Chinese automatic scoring model. In Table 6, the basic features (NN, NE, SN) based on graphs are positively correlated with the holistic score and the four essay trait scores, which are consistent with previous research results (Antiqueira et al., 2007; Amancio et al., 2012). The values of MI and MPR are statistically highly correlated with the main idea score, and similarity to the high-scoring essays has the highest correlation with the content score. This indicates that the similarity of the figures can reflect the similarity of the essay and provide a visualization of the essay; WDAP and WDNP have the highest correlations with the development scores, respectively, and this suggests that the distance between the concepts that combine the structure of graphs and the word vectors reflects the concepts transformation and essay development. The research results show that the model covers the three dimensions of the scoring rubrics, and the contribution of the graph-based features to predict holistic essay scores is stable on the datasets. It should be noted that the purpose of this study is still to predict the holistic score of an essay and focus on enhancing the coverage of writing constructs. Although automatic scoring models have been built for different essay traits because they have a high correlation with each other, the corresponding relationship between feature values and trait scores is still complicated. In the future, the scoring framework for individual potential traits will need specialized in-depth research.

Third, even if features based on language networks were used in previous automatic essay scoring studies (Ke et al., 2016; Somasundaran et al., 2016), the graph characteristic features were not integrated with word vector features. From a global perspective, graphs help enrich our understanding of the structure of the conceptual system organized by writers. Furthermore, the differences in the conceptual organization structure between excellent and poor writing can be identified, and a standard framework for automatic scoring can be established to pave the way for students to receive feedback on their writing strategies. From a local perspective, this study uses local-semantic node information based on word2Vec to calculate the distance between nodes. Table 6 shows that the value of MI is positively related to the main idea; WDAP and WDNP are positively related to the consistency score, and this indicates that they are closely related to the development level of the trait. This relationship can be seen and explained intuitively from the graph.

The present study has noteworthy limitations that should be discussed. In this study, word2Vec embeddings are used to represent the concepts in an essay; the word2Vec embeddings are not context dependent, but a static representation, making it not optimal for representing polysemous words, especially in Chinese. Polysemous words in mandarin Chinese happen at the character level, while the main body of modern Chinese contains phrases made up of more than one character, which may make the polysemy issue worse. More specifically, in determining the graph nodes from the composition, we hope to get concepts clustering rather than clustering of word forms. However, the clustering of concepts is divided into two different situations. First, when clustering, we must ignore the subtle semantic concept differences when it is used in different contexts and grasp the core concept semantics. For example, the word “teacher” can refer to the person, such as “teacher Zhang” in a composition, but it can also refer to the general term of this professional group. In this case, words with similar meanings should be grouped together and considered as the same concept. The second situation is the problem of distinguishing different concepts that share the same word form, which is called polysemy. Polysemous words carry more than two concepts, which occupy different positions in the semantic network and have different relationships with other concepts. Therefore, the identification and determination of concepts is an important part of this study, and it is closely related to the level of natural language processing technologies such as word segmentation and referential resolution. The concept recognition method adopted in this paper is a relatively compromised and simple method taken after considering both situations at the same time, but it may not be the optimal or most accurate method. In future research, it may be possible to narrow down the genre and topic scope of a writing task to construct a more specialized essay graph, so as to process and recognize the concepts in a certain corpus more precisely and accurately.

In addition, it should be noted that although the fairness test across subgroups was performed in this study, AECE is still trained and tested based on written data from eighth-grade Chinese students. We believe AECE is suitable for rating Chinese middle school students’ essays. However, it is necessary to be cautious in extending it to other grades or assessment projects, which might require further evidence of reliability and validity.

## Conclusion

This research examines a set of writing features deemed most relevant for the writing criteria of writing. This method proposed a new way to grasp global characteristics in Chinese writing by combining network analysis and traditional word-embedding technology, along with a proposed set of graph-based features corresponding with the essay scoring rubric to measure the quality of the main idea, content, and development. When comparing the proposed feature framework with the baseline feature set, the proposed feature framework outperformed the baseline and human raters, and it generated a higher accuracy score by using a multiple linear regression prediction for the middle school students’ essays. Furthermore, the feature framework extends the construct-relevant coverage of AES. By examining the relationship between the developments of potential traits in a student’s writing ability and the graph of that essay, there is a promise for a long-term cooperation between computational linguists and psychometric experts.

## Disclaimer

The opinions expressed are those of the author and do not represent views of the Institute or the Department of Education.

## Data Availability Statement

The datasets presented in this article are not readily available because the project is still ongoing and we do not have permission to share these data. Requests to access the datasets should be directed to xintao@bnu.edu.cn.

## Author Contributions

LY have made substantial contributions to the conception or design of the work; or the acquisition, analysis, or interpretation of data for the work, have drafted the work or revised it critically for important intellectual content. TX have design the research and the detailed method and processing, have drafted the discussion and conclusion part, CC have modified some important knowledge content of the article, including the clarification of the core concept within the article, the Data description in Method chapter, the illustration of word embedding, and have involved the text mining and feature selection process to ensure the accuracy and integrity of some content of the article, as well as the appropriate solution of related problems. All authors contributed to the article and approved the submitted version.

## Conflict of Interest

The authors declare that the research was conducted in the absence of any commercial or financial relationships that could be construed as a potential conflict of interest.

## Footnotes

1 The graph is a mathematical representation of a concept map with features that represent contextualized essay information.

2 LTP-Cloud, *See: http://www.ltp-cloud.com/intro_en*, developed by the Research Center for Social Computing and Information Retrieval at Harbin Institute of Technology (HIT-SCIR).

3 Big Cilin (V2.0). URL: http://www.bigcilin.com, it was compiled by Mei in 1983 and extended by HIT-SCIR (2018, 2019); it is a Chinese synonym database similar to the WordNet of English.

4 See the annotation of dependency relations of LTP: http://www.ltp-cloud.com/intro_en#dp_how

5 The size of the corpus is 4.1G, and the vocabulary size is 5,422 K; the embeddings trained on this corpus are at GitHub: https://github.com/Embedding/Chinese-Word-Vectors
