# Supplementary material for: Predicting Evaluations of Essay by Computational Graph-Based Features
Source: Front Psychol. 2020 Nov 12;11:531262. doi: 10.3389/fpsyg.2020.531262 (PMC7689217; doi:10.3389/fpsyg.2020.531262)
Supplement: Supplementary file 1 [file Data_Sheet_1.pdf]

Supplementary

Appendix A

Holistic scoring rubric for Chinese essays (prompt 1, prompt 2 and prompt 3)

Score	Scoring guide
6	Excellent response:<br>• takes a clear main idea, sustaining well-organized ideas effectively address the topic<br>• uses appropriate vocabulary, explanations, and detail to support the main idea, demonstrates facility with the conventions of standard written Chinese<br>• displays fluently expression and well developed with effective transitions and meaningful variety.
5	Skillful response:<br>• has a focused main idea and maintains generally connecting ideas appropriately<br>• supports the ideas with pertinent content through much of the response and conveys meaning clearly, occasionally uses words inaccurately<br>• exhibits some variety in sentences and progression of ideas but may lack some transitions.
4	Sufficient response (maybe marked by one or more of the following):<br>• takes an adequately focused main idea and addresses the topic well though some points may not be fully elaborated<br>• supports the ideas with some pertinent content and conveys meaning adequately, but may have some errors<br>• exhibits some coherence and develops the ideas to some extent, sentences and words may be simple and unvaried with few transitions.
3	Insufficient response (maybe marked by one or more of the following):<br>• attempts to take a position to addresses topic, but the position or main idea is unclear<br>• provides uneven support for the ideas and no development, or maybe very brief<br>• the response may be a short or too repetitive or accumulation of errors in lexical and usage that interfere with understanding.
2	Unsatisfactory response (maybe marked by one or more of the following):<br>• demonstrates poorly focused main idea and disorganized ideas, or demonstrates serious problems to express ideas<br>• provides few or irrelevant information in much of the response to address the topic, sentences, and word choice may often be inaccurate and interfere with understanding.<br>• The response is very undeveloped and using very limited and very simple vocabulary.
1	off-topic<br>• takes an unfocused main idea or position to address the topic<br>• provides no relevant content in response to the topic<br>• maybe very few words, or only a beginning and does not respond to the topic<br>• errors severely impede understanding the response
0	No answer (maybe marked by one or more of the following):<br>• is blank<br>• is illegible, nonverbal, or unmeaningful words.

Holistic scoring rubric for Chinese essays (prompt 4, prompt 5 and prompt 6)

Score	Scoring guide
4	Excellent response:<br>• takes a clear main idea, maintains generally connecting ideas appropriately<br>• uses appropriate vocabulary, explanations, and detail to support the main idea, demonstrates facility with the conventions of standard written Chinese<br>• exhibits fluently expression and well developed with effective transitions and meaningful variety.
3	Sufficient response (maybe marked by one or more of the following):<br>• takes an adequately focused main idea and addresses the topic well though some points may not be fully elaborated<br>• supports the ideas with some pertinent content and conveys meaning adequately, but may have some errors<br>• exhibits some coherence and develops the ideas to some extent, sentences and words may be simple and unvaried with few transitions.
2	Insufficient response (maybe marked by one or more of the following):<br>• attempts to take a position to addresses the topic, but the position or main idea is unclear<br>• provides uneven support for the ideas and no development, or maybe very brief<br>• the response may be a short or too repetitive or accumulation of errors in lexical and usage that interfere with understanding.
1	off-topic<br>• takes an unfocused main idea to address the topic<br>• provides no relevant content in response to the topic<br>• maybe very few words, or only a beginning and does not respond to the topic<br>• errors severely impede understanding the response
0	No answer (maybe marked by one or more of the following):<br>• is blank<br>• is illegible, nonverbal, or unmeaningful words.

Appendix B

The baseline features and explanations

Descriptive features	Explanation
Number of characters	Total number of text characters in the text
Number of words	Total number of words in the text
Percentage of one-character words	The ratio of the number of words consisting of one character to the total number of words
Percentage of two-characters words	The ratio of the number of words consisting of two characters to the total number of words
Percentage of three-characters words	The ratio of the number of words consisting of three characters to the total number of words
Percentage of more than four characters words	The ratio of the number of words consisting of four or more characters to the total number of words
Average number of strokes	The average stroke number of a single character in a full text. Chinese characters are made up of simple strokes. The strokes of a pen or pencil are the movements that you make with it when you are writing Chinese characters. (e.g., The character "天" (sky) has four strokes)
Low stroke ratio	The number of characters with 1-10 strokes divided by the number of full-text characters
Medium stroke ratio	The number of characters with 11-20 strokes divided by the number of full-text characters
High stroke ratio	The number of characters with more than 21 strokes divided by the number of full-text characters
Average number of words in a sentence	The total number of words in the text divided by the number of sentences
**Words features**	**Explanation**
Number of content words	The number of all content words in the full text
Number of function words	The number of all function words in the full text
Number of nouns	The number of all nouns in the full text
Number of verbs	The number of all verbs in the full text
Number of adjectives	The number of all adjectives in the full text
Number of adverbs	The number of all adjectives in the full text
Number of numerals	The number of all numerals in the full text
Number of quantifiers	The number of all quantifiers in the full text
Number of pronouns	The number of all pronouns in the full text
Number of prepositions	The number of all prepositions in the full text
Number of conjunctions	The number of all conjunctions in the full text
Frequency of content words	The logarithm frequency of content words in the full text
Frequency of function words	The logarithm frequency of function words in the full text
Frequency of nouns	The logarithm frequency of nouns in the full text
Frequency of verbs	The logarithm frequency of verbs in the full text
Frequency of adjectives	The logarithm frequency of adjectives in the full text
Frequency of adverbs	The logarithm frequency of adverbs in the full text
Frequency of numerals	The logarithm frequency of numerals in the full text
Frequency of quantifiers	The logarithm frequency of quantifiers in the full text
Frequency of pronouns	The logarithm frequency of pronouns in the full text
Frequency of prepositions	The logarithm frequency of prepositions in the full text

Frequency of conjunctions	The logarithm frequency of conjunctions in the full text
**Connectives features**	**Explanation**
Parataxis	The frequency of parataxis conjunctions
Progressive	The frequency of progressive conjunctions
Selective	The frequency of selective conjunctions
Succession	The frequency of succession conjunctions
Transition	The frequency of transition conjunctions
Hypothetical	The frequency of hypothetical conjunctions
Causal	The frequency of causal conjunctions
Condition	The frequency of condition conjunctions
Purpose	The frequency of purpose conjunctions
Total connectives	The frequency of total connectives conjunctions
**Lexical diversity features**	**Explanation**
TTR of content words	The ratio of the number of content word types to the number of tokens appearing in the full text
TTR of total words	The ratio of the number of word types to the number of tokens appearing in the full text
Average word length	The average word length in the full text
**Sentences features**	**Explanation**
Number of sentences	Total number of sentences in the full text
Average sentence length	The average number of characters in a sentence in the full text
Short sentence length	The number of characters in the shortest sentence in the full text
Long sentence length	The number of characters in the longest sentence in the full text
Average number of sentences per hundred words	The average number of sentences per hundreds words in a full text
Number of simple sentences	The number of simple sentences in the full text
Proportion of simple sentence	The ratio of simple sentences to all sentences in the full text
Number of compound sentences	The number of compound sentences in the full text
Proportion of compound sentence	The ratio of compound sentences to all sentences in the full text
The minimum edit distance between adjacent sentences	The average of the minimum edit distance between the words of adjacent sentences in the text (Modified the algorithm of Levenshtein (1965) to adapt to Chinese)
The minimum edit distance between words in the text	The average of the minimum edit distance between all words in the text (idem)
The minimum edit distance between adjacent parts of speech	The average of the minimum edit distance between parts of speech in adjacent sentences in the text (idem)
The minimum edit distance between the parts of speech in the text	The average of the minimum edit distance between all parts of speech in the text (idem)
Structural similarity of adjacent sentences	Similarity of adjacent sentences based on dependency structure (Li, 2003)
Similarity of sentence structure in the whole text	Similarity of all sentences based on the dependency structure (Li, 2003)
**Cohesion features**	**Explanation**
Content word overlap of adjacent sentences	The proportion of adjacent sentence pairs sharing one or more of the same content words to the total number of sentences

Content word overlap of all sentences	The proportion of all sentence pairs that share the same content word to the total number of sentences
Noun overlap of adjacent sentences	The proportion of adjacent sentence pairs sharing one or more of the same nouns to the total number of sentences
Noun overlap of all sentences	The proportion of all sentence pairs that share the same noun to the total number of sentences
Verb overlap of adjacent sentences	The proportion of adjacent sentence pairs sharing one or more of the same verbs to the total number of sentences
Verb overlap of all sentences	The proportion of all sentence pairs that share the same verb to the total number of sentences
**LSA features**	**Explanation**
LSA sentence adjacent	The average of the LSA cosine between adjacent sentences
LSA sentence all	The average of the LSA cosine between all sentences in the text
LSA verb Overlap	The average of the verb LSA cosine between adjacent sentences
LSA givenness versus newness	The indicator of the information (as opposed to new information) exists in each sentence in a text, as compared with the content of prior text information.

Appendix C

Graph construction from an essay

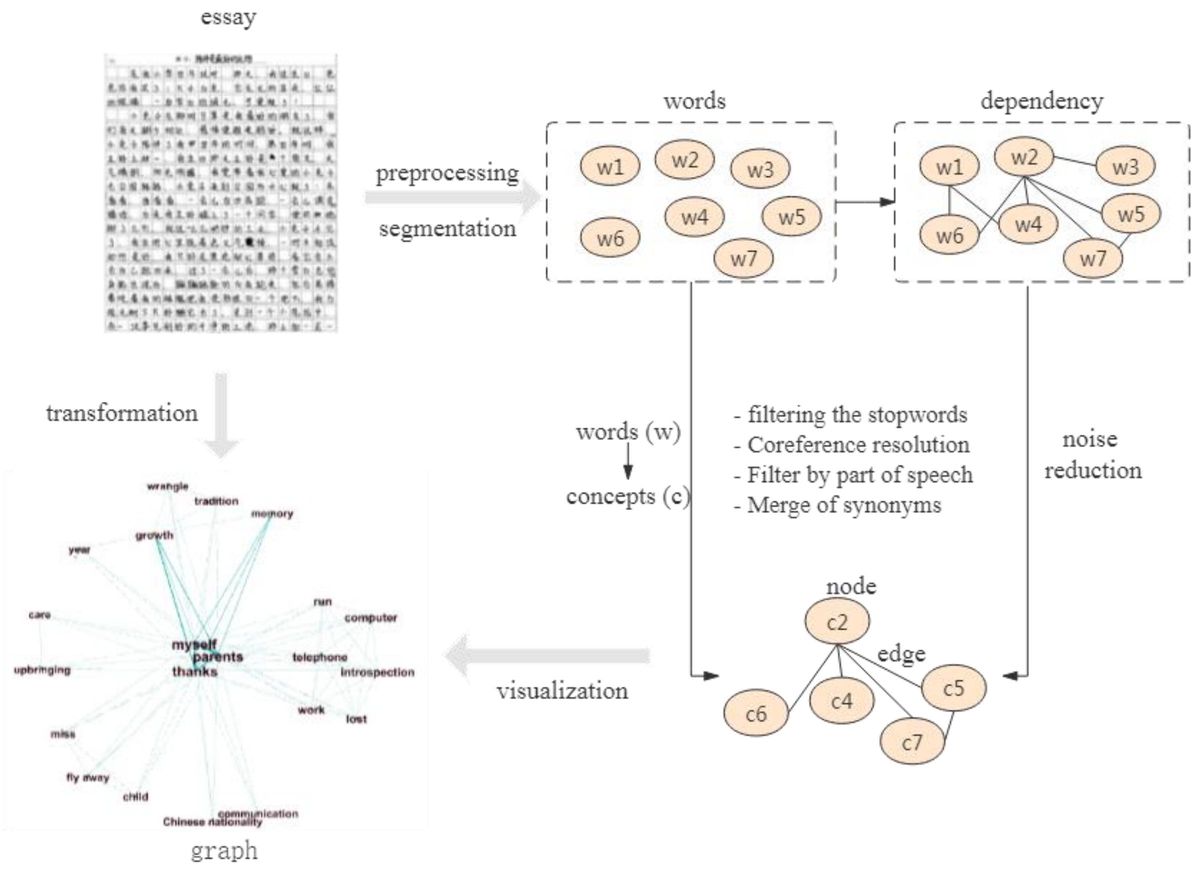

Note:

In practice, all words and dependencies in an essay are usually used to construct the initial graph. Then the concepts are identified, and only valid concepts and related edges are retained as the final graph.
